# Collective view: mapping *Sargassum* distribution along beaches

**DOI:** 10.7717/peerj-cs.528

**Published:** 2021-05-13

**Authors:** Javier Arellano-Verdejo, Hugo E. Lazcano-Hernández

**Affiliations:** 1 Department of Observation and Study of the Earth, Atmosphere and Ocean, El Colegio de la Frontera Sur, Chetumal, Quintana Roo, Mexico; 2 Cátedras CONACYT, El Colegio de la Frontera Sur, Chetumal, Quintana Roo, Mexico

**Keywords:** Crowdsourcing, Convolutional neural networks, Artificial intelligence, Beach monitoring, GIS, Ecological application, Crowd-mapping, Geotagged images

## Abstract

The atypical arrival of pelagic *Sargassum* to the Mexican Caribbean beaches has caused considerable economic and ecological damage. Furthermore, it has raised new challenges for monitoring the coastlines. Historically, satellite remote-sensing has been used for *Sargassum* monitoring in the ocean; nonetheless, limitations in the temporal and spatial resolution of available satellite platforms do not allow for near real-time monitoring of this macro-algae on beaches. This study proposes an innovative approach for monitoring *Sargassum* on beaches using Crowdsourcing for imagery collection, deep learning for automatic classification, and geographic information systems for visualizing the results. We have coined this collaborative process “Collective View”. It offers a geotagged dataset of images illustrating the presence or absence of *Sargassum* on beaches located along the northern and eastern regions in the Yucatan Peninsula, in Mexico. This new dataset is the largest of its kind in surrounding areas. As part of the design process for Collective View, three convolutional neural networks (LeNet-5, AlexNet and VGG16) were modified and retrained to classify images, according to the presence or absence of *Sargassum*. Findings from this study revealed that AlexNet demonstrated the best performance, achieving a maximum recall of 94%. These results are good considering that the training was carried out using a relatively small set of unbalanced images. Finally, this study provides a first approach to mapping the *Sargassum* distribution along the beaches using the classified geotagged images and offers novel insight into how we can accurately map the arrival of algal blooms along the coastline.

## Introduction

Studies have demonstrated the negative impact of large concentrations of *Sargassum* along the coast and beaches on existing ecosystems. Some examples include: enhanced beach erosion (Van Tussenbroek et al., 2017), mortality of near-shore seagrass and fauna due to *Sargassum* leachates and abundant suspended organic matter that obstructs the passage of light to deeper areas of the coast (Rodríguez-Martínez et al., 2019; Van Tussenbroek et al., 2017), and the alteration of the trophic structure of the sea urchin Diadema antillarum along coastal marine systems (Cabanillas-Terán et al., 2019). Additionally, it is known that onshore and nearshore masses of *Sargassum* interfere with the seaward journeys of the juvenile turtles, affecting their nesting (Maurer, De Neef & Stapleton, 2015). Finally, the high arsenic content present in *Sargassum* is of concern for environmental contamination of the sea and aquifer. In the study of Rodríguez-Martínez et al. (2020), 86% of the total samples contained arsenic concentration amounts exceeding the maximum limits for seaweed used as animal fodder (40 ppm DW). Thus, the authors recommended instrumenting obligatory practices of metal content analyses in *Sargassum* or avoiding the use of it for nutritional purposes. As demonstrated in these studies, there is sufficient scientific evidence that confirms the negative impact of large volumes of *Sargassum* on the coastal ecosystems of the Caribbean Sea. However, what seems to be missing from this body of research is how technological solutions (e.g., the use of an adequate spatial and temporal scale for regional and local monitoring) can contribute to the management and disposal of *Sargassum* along the beaches.

Since 2011, the massive arrival of pelagic *Sargassum* has altered the balance of coastal ecosystems in the Caribbean Sea (Gower, Young & King, 2013). Although *Sargassum* is of great ecological advantage in the open ocean, the high concentrations present in the coastal zone have generated ecological and economic damage (Van Tussenbroek et al., 2017). The Mexican Caribbean coastline began receiving massive amounts of *Sargassum* during late 2014, reaching its highest peak in September 2015 (Rodríguez-Martínez, Van Tussenbroek & Jordán-Dahlgren, 2016). During 2016 and 2017, the influx of *Sargassum* slowly declined and then increased again in 2018 (Rodríguez-Martínez et al., 2019). In 2019, the arrival of *Sargassum* continued, but with lower amounts than in 2018. A similar trend can be seen in the year 2020, according to the Outlook of 2020 *Sargassum* blooms in the Caribbean Sea (Hu et al., 2016). The amount of *Sargassum* approaching the coastline seems to be lower in 2020, when compared to 2019 (Hu et al., 2016). Despite the gradual decrease in the yearly amount of *Sargassum* arriving to the shores, one should be careful not to underestimate the continued effects. The total amount of *Sargassum* is still very high and continues to critically damage the ecosystem (Rodríguez-Martínez et al., 2019).

Traditionally, the monitoring of *Sargassum* has been carried out using satellite remote sensing techniques. The Terra, Aqua, and Landsat platforms have been the most widely used due to their sensors onboard, their technical features, and for providing open data (Wang & Hu, 2020). However, because of the temporal and spatial resolution limitations, it is not possible to monitor *Sargassum* efficiently with the open access satellite platforms previously described (Wang & Hu, 2020). Important methodologies for detecting *Sargassum* have been developed by inputting the data obtained from the aforementioned platforms. Currently, the most widely accepted remote sensing methodologies worldwide for detecting pelagic *Sargassum* are the Floating Algae Index (FAI) (Hu, 2009) and Alternative Floating Algae Index (AFAI) (Wang & Hu, 2016). However, the frequent presence of clouds in the region is an issue that causes false positives. Additionally, these methodologies do not offer precision in nearshore waters that are relevant to the local communities where the ecological and economic challenges occur (Wang & Hu, 2020). Recent research has aimed to monitor *Sargassum* along the coastline using computing science paired with remote sensing data. One example is ERISNet, which uses a new artificial neural network architecture to classify geospatial dataset values related to the presence or absence of *Sargassum* across various spectral bands (Arellano-Verdejo, Lazcano-Hernandez & Cabanillas-Terán, 2019; Álvarez-Carranza & Lazcano-Hernández, 2019). A second example is the use of a convolutional neural network (CNN) for automatic classification of Moderate Resolution Imaging Spectroradiometer (MODIS) satellite products, enabling high-generalization classifications of more than 250,000 images with a 99.99% accuracy (Arellano-Verdejo, 2019). Finally, Wang & Hu (2020) conducted an innovative study where *Sargassum* features were automatically extracted from Sentinel-2 MSI Images (Wang & Hu, 2020). One of the shortcomings of these studies can be found in the resolutions of the sensors. The data obtained from the MODIS sensor, which is onboard Terra and Aqua platforms, has a spatial resolution between 250 m and 1.2 km, depending on the spectral band, with a revisit time of one day. On the other hand, the MSI sensor onboard Sentinel-2 offers a spatial resolution of 10, 20, or 60 m depending on the spectral band. The revisit frequency of each single Sentinel-2 satellite is ten days and the combined constellation revisit is five days. Therefore, limitations in the spatial (MODIS) and temporal (MSI) resolutions of the sensors are still present.

Another approach that emerged recently for monitoring *Sargassum* involves the use of social networks. However, the lack of metadata in the images makes this approach ineffective (Uribe-Martnez et al., 2020). Other initiatives, inspired by the use of “Citizen Science” and “crowdsourcing”, have collected information on the arrival of the *Sargassum* to the coast (Arellano-Verdejo & Lazcano-Hernandez, 2020). These initiatives have offered promising results as well as new challenges. The term crowdsourcing was coined by Howe (2006), to describe “the act of taking a job traditionally performed by a designated agent (usually an employee) and outsourcing it to an undefined, generally large group of people in the form of an open call” (Howe, 2006). Several studies have demonstrated that Crowdsourcing is a useful methodology for collecting and managing data in different content areas, including: image classification, character recognition, genome annotation, document translation, protein folding, RNA structure design, algorithm development (Good & Su, 2013), and *Sargassum* monitoring (Arellano-Verdejo & Lazcano-Hernandez, 2020). For the successful implementation of crowdsourcing, it is advisable to use a platform to standardize and automatize the study processes. In this sense, there are two possible ways: to use an existing platform or, if necessary, to create a platform according to the goals and context of the study. There are important Citizen Science platforms that can be used to collect information (e.g., iNaturalist and Epicollect). iNaturalist (https://www.inaturalist.org/), managed by the California Academy of Sciences and the National Geographic Society, is a Citizen Science project and online social network of naturalists, scientists, and biologists. This application maps and exchanges biodiversity observations around the world. The main advantages of this platform are that the data is openly accessible on the Web and that much of it is licensed for re-use or free of intellectual property restrictions. Epicollect (https://five.epicollect.net/), managed by the Big Data Institute of the University of Oxford, is a very robust platform in which, in addition to images, different data can be captured in text format through its user interface. One of the disadvantages of this platform is the design. Intended for an expert user (i.e., scientists, technicians, and students), it becomes complex for someone who is not familiarized with the topic. Applications such as iNaturalist and Epicollect are designed to measure species presence/absence rather than abundance estimation or surveillance. However, the present study requires periodic monitoring over time rather than sporadic sightings, which means photographs are also required, even when there is no *Sargassum* on the beach. Therefore, we chose to build a platform according to the goals and context of the study region.

The creation of new neural network architectures in recent years has enabled their use in massive commercial applications (Abiodun et al., 2018). Some examples include: Adversarial Generative Networks (GANs), Autoencoders, Residual Networks (ResNets) and Unets. However, Transfer Learning, defined as taking previously trained neuronal models and adapting them to different problems for which they were created, has solved problems like the one presented in this work, both in a timely and successful manner. In addition, increased storage capacity and the emergence of new massive processing technologies, including GPU and TPU clusters, have dramatically increased the processing power we have nowadays. This has led to an increase in research employing the development of new algorithms of Artificial Intelligence (AI) and Machine Learning (ML), resulting also in the design and effective operation of various applications, as mentioned previously (Abiodun et al., 2018).

Maps are essential resources for visitors to an unfamiliar place because they visually highlight landmarks and other places of interest. However, hand-designed maps are static representations that cannot always be adapted to the study of the phenomenon of interest, especially when the changes in the phenomenon are highly dynamic. In the last decade, digital maps, such as those provided by Google Maps, Waze Maps, Uber, and Rappi, have become increasingly popular (Graaf, 2018). Many phenomena are usually in a perpetual state of change and renewal. Therefore, one of the advantages of these digital maps over hand-designed maps is that they are based on continuously updated models and generally reflect the most current information (Grabler et al., 2008). Modern earth-observation research aims to study the variation in landscapes over multiple spatial and temporal scales. As mentioned previously, there is not always availability of satellite data on land coverage, so the combined use of crowdsourcing, mobile telecommunications, and the internet has become a viable solution. In this sense, previous research has generated the crowdmapping of urban objects with geo-location precision of approximately 3 m (Qiu et al., 2019) and land use maps in big cities via Twitter (Frias-Martinez & Frias-Martinez, 2014). In addition, based on photographs uploaded to social networking sites, there are studies that generate maps for evaluating the flow of services of the cultural ecosystem (Karasov et al., 2020). These few samples show that map generation, through crowdsourcing, is a reliable methodology that has been implemented successfully in several studies.

In terms of *Sargassum* monitoring, research points to important ocean scale mapping efforts. Some examples include: the *Sargassum* Early Advisory System (SEAS), developed by Texas A&M University at Galveston (Webster & Linton, 2013), and the Satellite-based *Sargassum* Watch System (SaWS), at the University of South Florida (USF). The latter relies on near-real-time satellite and modeling results to monitor pelagic *Sargassum*, which serve to create monthly bulletins and show the distribution maps in the central-west Atlantic Ocean and Caribbean regions (Duffy et al., 2019; Hu et al., 2016). Other tools have been developed to integrate SaWS products for visualization using Google Earth, which facilitates the application of SaWS products through a widely known visualization tool (Maréchal, Hellio & Hu, 2017). Finally, “Citizen Science” platforms, such as Epicollect and iNaturalist, allow groups of researchers to build sets with geo-referenced images of *Sargassum*. In this sense, the Marine Macroalgae Research lab at Florida International University (MMRL-FIU) is studying the occurrences of washed-up *Sargassum* landings on South Florida and Caribbean coastal areas, through the crowdsourcing project called “*Sargassum* Watch”. This project can be accessed through the iNaturalist and Epicollect platforms. At the end of 2020, the *Sargassum* Watch project had collected 980 observations using iNaturalist (https://www.inaturalist.org/projects/sargassum-watch-inaturalist-version) (carried out by 577 people) and over 2,155 photographs using the Epicollect (https://five.epicollect.net/project/sargassum-watch/data) platform. On the other hand, in Mexico, the National Commission for the Knowledge and Use of Biodiversity (CONABIO, for its acronym in Spanish) manages the project “Monitoring pelagic *Sargassum* in the Mexican Atlantic”, which can be accessed through the Naturalista platform (https://www.naturalista.mx/projects/sargassum-watch-inaturalist-version). At the end of 2020, CONABIO’s project had collected 154 observations, carried out by 50 people. Photographs included both close-up and panoramic shots of *Sargassum*. Despite these important efforts to monitor *Sargassum* along the beaches, the diversity of images (i.e., visual attributes like angle, background elements, lighting, lens distortion) for the region of the study is limited. Thus, the number of attributes shown in the images collected with these applications is limited in our study. However, this is not unusual given the purpose for which these applications were originally created. They were initially used to integrate a database that contributes to the knowledge of the biodiversity of the species, and in particular with *Sargassum*, to know the distribution of this macroalga throughout the Caribbean. Furthermore, there is not a constant flow of photographs uploaded to the platforms by citizens. Another limitation is that, in order to produce useful tools for decision-making processes on how to manage and dispose *Sargassum*, it is necessary to have an automatic classification algorithm of the imagery, which can generate automatic *Sargassum* distribution maps. To the best of our knowledge, there is no system, at the beach scale, that automatically generates maps regarding the presence or absence of *Sargassum*. A tool of this type will allow organizing geotagged photographs at scales of less than one meter, for the construction of a collaborative network that complements remote sensing observations. The monitoring of *Sargassum* on beaches is a great challenge.

This study proposes a collaborative scheme based on crowdsourcing for the capture of images, artificial intelligence algorithms for the automatic classification of photographs, and geographic information systems for displaying the results. This collaborative process has been called “Collective View”, which is a living process that, due to the social collaboration, currently continues generating geotagged images along the northern and eastern coasts of the Yucatan Peninsula. Because crowdsourcing relies on the contribution of society, we used several strategies to involve individuals in the monitoring of *Sargassum* on the beach. Some of these strategies included: lectures given at numerous institutions in the region, direct invitations sent to academics and students interested in *Sargassum* issues, and the creation of a short video explaining how to use the app “Collective View” (https://www.youtube.com/watch?v=201Du7pJ0g8). Therefore, we consider this study contributes to improving *Sargassum* monitoring along the beach and supports its management and disposal. The actions carried out in each stage of the “Collective View” design process are described in detail below.

## Materials and Methods

Following a crowdsourcing paradigm, the software that compose “Collective View” were designed, developed, and implemented for the acquisition, storage, and processing of geotagged photographs. The software program combines three main components: a mobile application for smartphones with Android operating system (https://play.google.com/store/apps/details?id=appinventor.ai_javier_arellano_verdejo.ERIS_SMShl=es). Currently, the “Collective View” application is available for Mexico, Belize, Colombia, Guadalupe Island, and Florida (USA). This app was designed to acquire images, the coordinates of the mobile device, and other data from the phone sensors (e.g., accelerometer values, gyroscope) that help to know the phone’s attitude when a picture is taken. The overall objective of the App is to have an easy-to-use tool that stores information in the cloud simply and transparently for the user.

The data collected by the App is sent and stored in the cloud using Google’s firebase service. Although there are other tools, the purpose of using firebase is to serve as temporary storage of the information collected by users and to manage high bandwidth, as well as multiple simultaneous requests quickly and uninterruptedly. At the same time, we have a backend service that continually analyzes the data stored in the cloud and synchronizes them with our central servers, responsible for classifying and analyzing the information provided by users. All of this is done using the Python programming language and the PyTorch library running on a Lenovo workstation with an Intel Xeon EP processor, 64 GB of RAM, NVidia Quadro K5000 GPU running the Linux operating system Ubuntu 18.04 64 bits. Details and versions of all libraries are provided in a file in the Supplementary Material.

**Figure 1:**
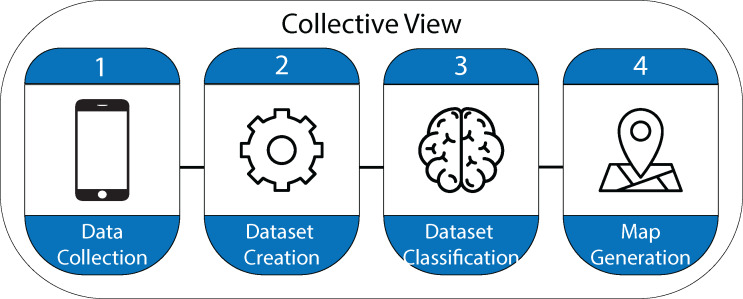
Stages in the design process of the Collective View ecosystem.

Once the data is analyzed and processed, a geographic information systems compatible shapefile is created containing the points where the images were taken. The information related to those points is visualized in the form of maps. A dashboard was designed using Arcgis Online to visualize the information, providing the user with different layers of information. Figure 1 shows each of the stages followed in the process of designing the Collective View ecosystem.

### Data collection

This study was carried out in the northern (from 21.247893, −89.834655 to 21.357756, −89.115214) and eastern regions (from 21.233803, −86.801127 to 18.266237, −87.835622) in the Yucatan Peninsula, in Mexico. This area is composed of beaches where massive arrivals of *Sargassum* have been recorded in recent years (2015, 2018–2020).

**Figure 2:**
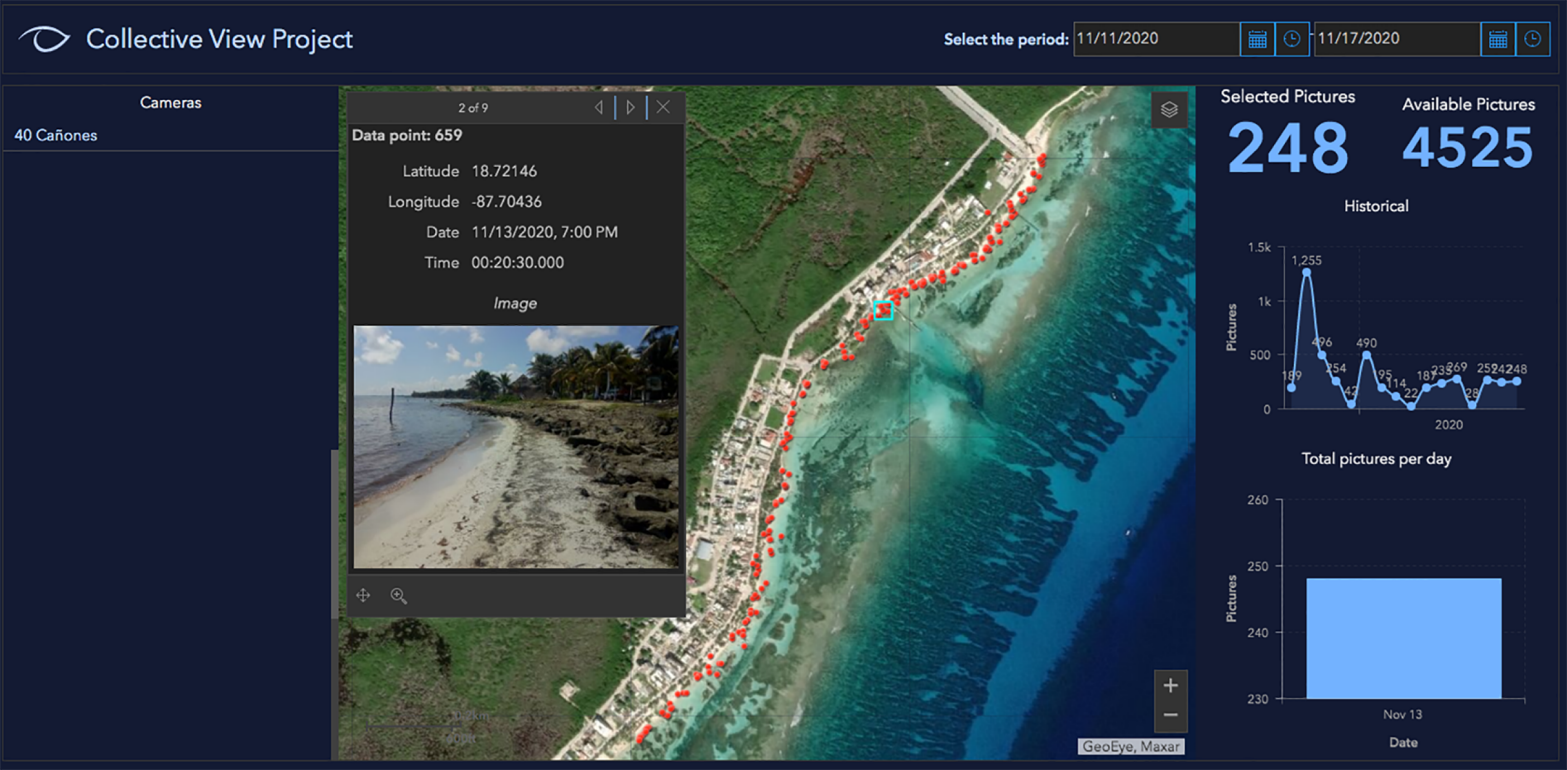
Dashboard in Collective View.

The images gathered to build the dataset of the present study were collected through the crowdsourcing mobile app of “Collective View”. Due to the origin of the photographs, their quality is beyond our control, and may depend on users’ skills to take pictures, the environmental conditions and the device’s features (i.e., the sensor size, resolution in megapixels, etc). However, the variety of photographs (i.e., angle, background elements, lighting, lens distortion), contributes to the neural network training and improves its performance, photographs out of focus or with poor lighting also contribute to the training of the neural network. That is why it is desirable to have photographs with different characteristics. On the other hand, the information in a photograph has its own limits; a greater amount per area, per day, will allow us to achieve the best results. However, this analysis is beyond the scope of this study. These images enabled researchers to compile geotagged photographs regarding the presence or absence of *Sargassum* along the beaches in the Yucatan Peninsula. The geotagged photographs that individual members of society upload to the platform can be viewed through a web dashboard. At the time of conducting the experiment, the Collective View dataset had 4,525 photographs of eleven cities in the states of Yucatan and Quintana Roo. This reveals that Collective View offers the biggest dataset worldwide of the presence or absence of *Sargassum* along the beaches in this region. Sixty-two users participated in the construction of this data set. The images collected through the Collective View app include metadata regarding the latitude and longitude of the place where each photograph was taken, the date, the hour with minutes and seconds, as well as additional data from the gyroscopes, accelerometers, and other sensors of the smartphone. At the time of building the dataset for the training stage, the number of images on the platform was lower, therefore the sum of images of the four instances is less than the total number of images. Crowdsourcing dataset features are shown in Table 1. Currently, geotagged photographs can be consulted on the internet (https://arcg.is/1STq0C). Figure 2 shows an image of the online dashboard.

**Table 1:** Crowdsourcing dataset feature.

Crowdsourcing dataset features	
Number of images	4,525
Number of classes	4
Number of instances with *Sargassum*	647
Number of instances without *Sargassum*	1,012
Number of instances with other algae	1,645
Number of instances with other elements	25
Metadata of the photograph	lat, long, date, hour, minute, second
Number of cities along the beach	11
Number of states in the Yucatán Peninsula	2
Number of participants in the Crowdsourcing activity	62

The next step followed in the data collection involved the use of crowdsourcing information to generate automatic maps illustrating the accumulations of this macroalgae. These maps could also be used by citizens and tourists to know the conditions of the beaches, either for decision-making processes or simply for planning a trip. To generate these products from the collected images, two requirements needed to be met: first, the platform needed to receive a constant flow of information; and second, images needed to be classified automatically. To ensure the first requirement was met, we widely disseminated the crowdsourcing campaign and carried out various activities to raise awareness in society. It is important to mention that this requirement can pose different challenges across cultures, depending on their habits and customs, and is outside of the scope of this study. The following section describes the second requirement, the methodology used for the automatic classification of images with the presence or absence of *Sargassum*.

### Dataset creation

The following sections describe the processes followed to build the image data set, which was later used to train the different neural network architectures for classifying images illustrating the presence or absence of *Sargassum*. The initial dataset used for the training, testing, and validation of the neural networks was classified manually by experts in the subject of study.

#### Training and validation dataset

To analyze the accumulation of *Sargassum* on the beaches in Quintana Roo and generate maps to show its distribution, we had to train and select the hyper-parameters of the neural network. This was an essential step of the process that allowed us to classify images according to the presence and absence of *Sargassum*. However, it is also one of the parts of the process that presented multiple challenges.

The lack of geotagged *Sargassum* images dataset was one of the main obstacles to overcome. In order to address this issue, we integrated a set of 2,400 images stemming from several sources of information. A total of 1,720 geotagged images were collected through Collective View, as described above Other images were collected using the Google image search engine. We conducted a search of historical images for the coasts of Quintana Roo in recent years, originating a total of 600 images, which were downloaded from the internet. Finally, 80 pictures were taken with a traditional digital camera and were added to the dataset. Although these images were not geotagged, they were used only during the training, testing and validation processes of the neural network.

Within the 2,400 images collected, 1,200 exhibited the presence of *Sargassum* and 1,200 images did not contain the presence of *Sargassum*. The dataset of images used to train the neural network, was classified by hand by experts in the field in order to ensure that the images are correctly classified and thus to train the neural network. For the validation of the neural network, we randomly selected 20% of the images (480), from which 50% contained images with *Sargassum* presence, and the remaining 50% of images without *Sargassum* presence. The remaining 80% of the original dataset of images (1,920) was randomly divided again into two subsets. The first subset contained 80% of the images (1,536), which were used for the training process. The remaining 20% of images (384) were used to test the network during the same training and hyper-parameter selection process. As one can observe, the dataset used to train the neural network is too small when compared to the classical data sets. This represented a challenge for training the neural network without falling prey to overfitting. In order to allow for reproducing the results obtained in this study, the images were stored maintaining the structure mentioned above.

**Figure 3:**
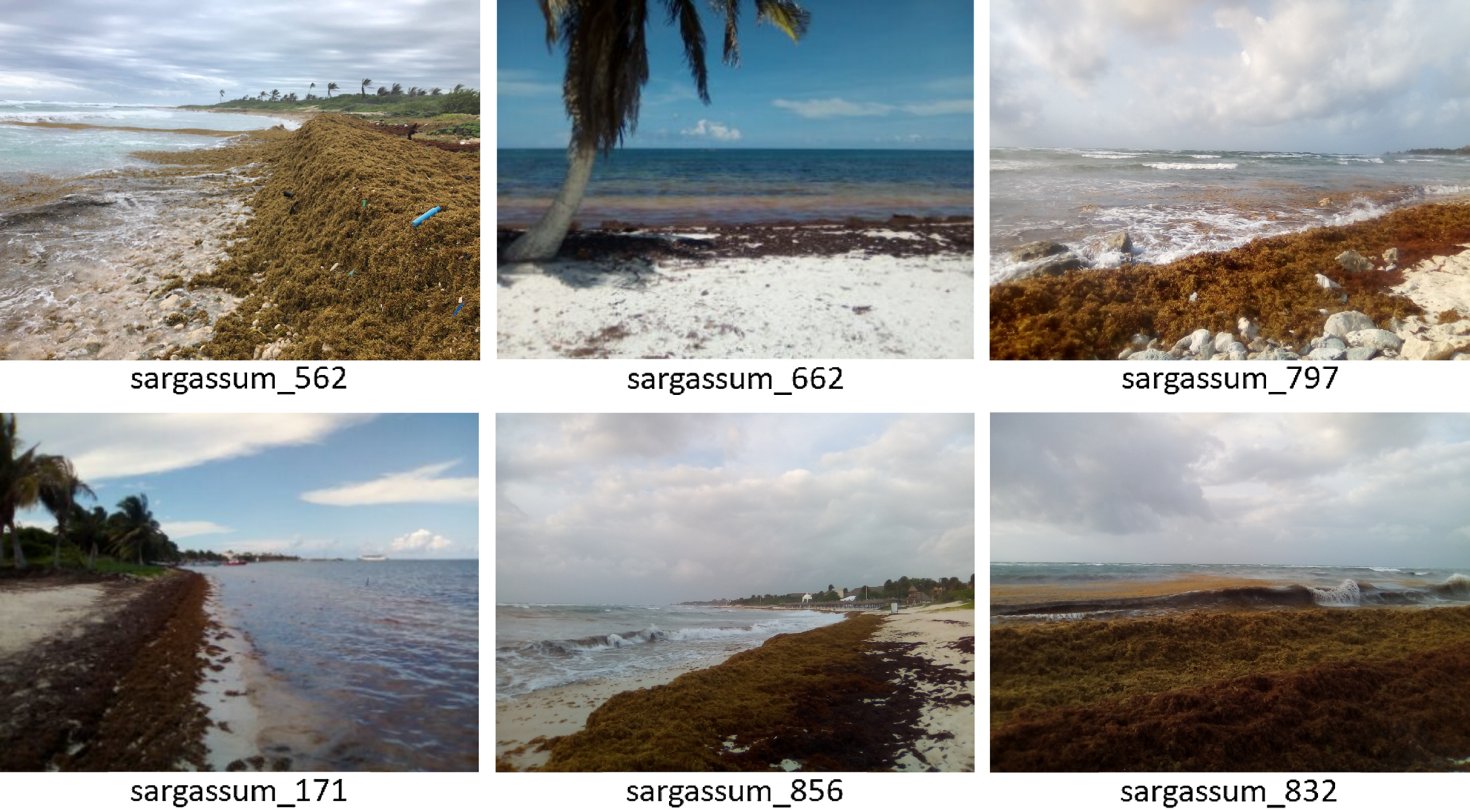
Sample photos of the dataset, (located in the “train/sargassum” folder). The dataset are available for download in the link: https://doi.org/10.6084/m9.figshare.13256174.v5.

Figure 3 shows an example with six *Sargassum* images that form part of the dataset we have built. As demonstrated in the images, there is extensive variation in the features, angles, lighting, and other characteristics of the images. This causes the classification process to be even more challenging. Augmented Data and Transfer Learning methodologies were used to address this challenge, which in turn, decreased overfitting and improved network generalization.

#### Image data augmentation

Convolutional neural networks (CNN) and Deep Learning (DL) have been proven effective for image recognition as well as data processing. These techniques are based on supervised learning algorithms, so their effectiveness is strongly related to the quality and size of the datasets used for the training and validation of the models.

Nowadays, there are different open access datasets available. Most of them are mainly used for research purposes. However, many others are part of competitions that are carried out by big companies (e.g., Google and Netflix). Data sets such as MNIST, CIFAR10, ImageNet and COCO have been widely used as benchmarks to train, test, and compare new models with previous findings. One of the most relevant features of these datasets is their size. For example, MNIST used 60,000 images for training purposes and another 10,000 for the validation. Other models, such as COCO, likely have millions of data entries available.

Applying DL techniques to real problems presents multiple challenges. Since the data set is small, the diversity in the images is low, which reduces the amount of information available to the neural network during training. This will most likely cause the network to attempt to memorize the data rather than learn to generalize it (overfitting). There are multiple techniques that can be used to reduce overfitting. Some of them include dropout, batch normalization, and augmented data (Srivastava et al., 2014), (Ioffe & Szegedy, 2015), (Shorten & Khoshgoftaar, 2019). Since the size of the dataset for training the neural network is small compared to traditional datasets, we have used two strategies to deal with this challenge; on the one hand we have used augmented data to increase the number of images in the dataset and on the other hand we have employed transfer learning to use previously trained networks with large datasets, which increases the generalization capability of the neural network (Shorten & Khoshgoftaar, 2019).

Augmented data offers the possibility of dynamically transforming the input data. Some of the most common transformations performed include: flipping the images, zooming in/out of certain areas, random cuts, angle rotation, varying the amount of illumination and image contrast, among others (Shorten & Khoshgoftaar, 2019). The models presented in this paper were trained and validated using augmented data. The main adjustments to the data involved a random horizontal flip, a random rotation of 10 degrees, and a variation in the parameters of brightness, contrast, and saturation. Due to the use of augmented data, the accuracy of the network is improved while overfitting is controlled for.

**Figure 4:**
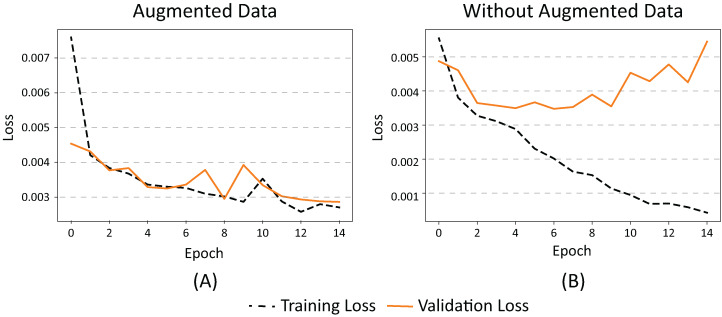
(A) Effects on error value when using augmented data in a CNN and (B) without augmented data.

The two images in Fig. 4 graphically demonstrate the effect of using augmented data during the training phase (epochs) in our dataset. When using augmented data (Fig. 4A), the loss (error) between the results of the training data classification and the validation data is similar.

When no augmented data is used (Fig. 4B), the classification error of the training data decreases while the validation error increases, which means that the network has started to memorize the noise from the images. This causes their generalization to decrease, resulting in overfitting. In summary, we can conclude that the use of augmented data in our dataset promotes the generalization of the neural network by maintaining a balance between error and overfitting.

### Dataset classification: convolutional neural networks

The following sections describe the different Neural Network architectures used to classify the dataset. We tested a total of three classic models. The first one consisted of using an adapted LeNet-5 CNN. The other two involved Transfer Learning, where two pre-trained models were adapted, namely the AlexNet and the VGG16 neural networks.

#### LeNet-5

**Figure 5:**
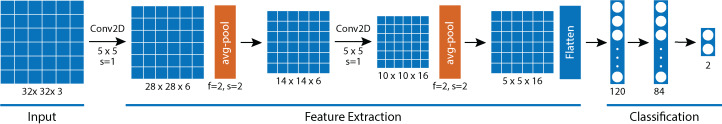
LeNet-5 Architecture.

LeNet-5 (Fig. 5) was developed In 1989 by LeCun et al. (1998) and was one of the earliest CNN. By modern standards, LeNet-5 is a very simple network, consisting of: two sets of convolutional and average pooling layers, a flattening convolutional layer, two fully-connected layers, and a softmax classifier. Initially, LeNet-5 was used on a large scale to automatically classify hand-written digits on bank cheques in the United States.

Comparable to virtually all CNN-based architectures, LeNet-5 encompasses two phases, a feature extraction segment and a classification segment. Figure 5 illustrates both phases. First, given an input image of 32 × 32 pixels, it uses a convolutional layer with a filter size of 5 × 5 and stride of 1. This generates a total of 6 attribute filters of 28 × 28 pixels each (28 × 28 × 6). To obtain the relevant information of each one of the generated filters, the output is processed by an Average Pooling operation (avg-pool). Using a filter size of 2 × 2 and stride of 2, the avg-pool generates a decrease in the size of the input from 28 × 28 to 14 × 14 pixels. In the next step, a convolutional operation and avg-pool are again applied to produce a 5 × 5 × 16 output.

The second phase corresponds to the classification of the extracted attributes. Since the input of the next phase is a fully connected layer, and the output of the attribute extraction phase is a set of 5 × 5 × 16 filters, a reshaped flattened operation is used for the input elements.

During the attribute classification phase, the last layer of LeNet-5 was modified to use two neurons (one for images with *Sargassum* presence and one for those without *Sargassum* presence). Thereby, the attribute classification phase was assembled with a fully connected network composed of three layers of 120, 80 and 2 neurons respectively.

Finally, it should be noted that all layers of the model, except the last one, used the hyperbolic tangent activation function. The last layer used the softmax function. For the training process, an Adam optimizer was employed with a learning rate of 0.001. The Cross-Entropy loss function was used.

#### AlexNet

Deep CNN models may take days or even weeks to train using large datasets. One way to reduce the length of this process is to re-use the model weights from pre-trained developed models for standard computer vision benchmark datasets (i.e., the ImageNet image recognition tasks). An assumption in many machine learning and data mining algorithms is that the training and future data must be in the same feature space and have the same distribution. However, in many real-world applications, like in the *Sargassum* image classification presented in this study, this may not remain true. In deep learning, transfer learning (Pan & Yang, 2009) is a technique where a neural network model is trained first using a sample dataset, similar to the one that represents the problem for which it is being created. Then, one or more layers from the trained model are used (learning transfer) in the training of the new model which responds to the real problem.

**Figure 6:**
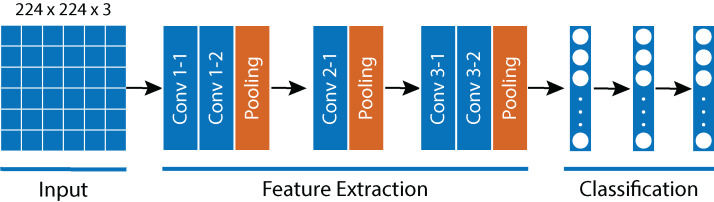
AlexNet Architecture.

AlexNet (Fig. 6) has 60 million parameters and 650,000 neurons. It contains eight learned layers. Among these eight layers, we find five convolutional layers, some of which are followed by max-pooling layers; and three fully connected layers, using the ReLu activation function. We used the PyTorch library (Paszke et al., 2019) and the Python programming language to lock all AlexNet attribute extraction layers. We also modified the last classification layer, which enabled us to adapt the output of AlexNet to the characteristics of our dataset. Through the application of augmented data and transfer learning, we were able to classify our dataset. For the training process, an Adam optimizer was employed with a learning rate of 0.0001. The Cross-Entropy loss function was used, and a Batch Size of 100 images for each epoch was employed. A total of 80% of the dataset was randomly selected for the training phase, while the remaining 20% was used during the validation phase. As demonstrated later in this section, we used Google Colab, as well as an execution environment using GPUs as processing units, to perform the AlexNet training. This resulted in nearly 90% accuracy, maintaining an adequate balance between the error and the overfitting.

#### VGG16

The VGG16 is a CNN model proposed by K. Simonyan and A. Zisserman, from the Visual Geometry Group (VGG) at University of Oxford in 2014 (Simonyan & Zisserman, 2014). The VGG16 was created with the purpose of enhancing classification accuracy by increasing the depth of the CNNs. The 16 in the VGG16 name (or 19 in the case of the VGG19) refers to the number of layers. In this case, the VGG16 model has 16 layers with trainable parameters. This network is quite large and comprises approximately 138 million of parameters in all. The VGG16 is considered one of the best computer vision models to date. What is unique about the VGG16 is that, instead of containing a large number of hyper-parameters, it is composed of convolution layers with very small filters.

**Figure 7:**
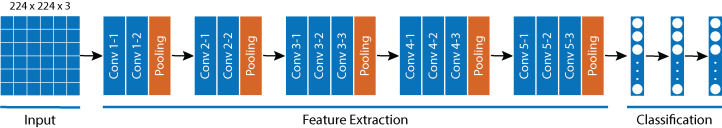
VGG16 Architecture.

The input layer of the VGG16 is an RGB image of 224 × 244 pixels. The image is processed by a stack of convolutional layers with the fixed filter size of 3 × 3 and stride of 1. There are five max pooling filters embedded between the convolutional layers in order to down-sample the input representation. The stack of convolutional layers is followed by three fully connected layers, consisting of 4,096, 4,096 and 1,000 channels, respectively. The last layer is a soft-max layer (Fig. 7).

Similar to the process carried out with AlexNet, we modified the last classification layer of the VGG16 to adapt it to the number of classifications in our dataset. We employed transfer learning, a model previously trained for ImageNet. All feature extraction layer weights were locked, so during the training phase, only the classification layer weights were retrained. Augmented data for the training process was used as well as the Adam optimizer, with a learning rate of 0.001. The Cross-Entropy loss function was used.

## Results and discussion

To classify the image dataset, we used three types of convolutional neural networks: LeNet-5, AlexNet, and VGG16. Each of them were adjusted for the classification process using our dataset. LeNet-5 was modified to work with RGB (three-band) images. The last layer of 1,000 categories in the AlexNet and the VGG16 architectures was replaced by a fully-connected layer for two classes (*Sargassum* and non-*Sargassum* images). Finally, for both the AlexNet and the VGG, we used augmented data and pre-trained models (transfer learning) to maximize the generalization capacity of the network for the given dataset.

**Figure 8:**
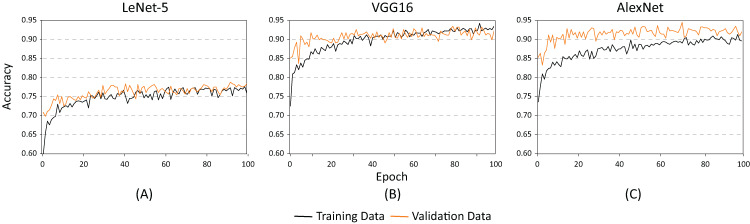
Accuracy results for batch size = 20 and learning rate = 0.001 for (A) LeNet-5, (B) VGG16, and (C) AlexNet.

**Figure 9:**
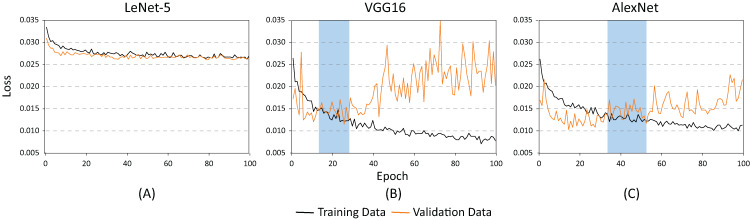
Loss results for batch size = 20 and learning rate = 0.001 for (A) LeNet-5, (B) VGG16, and (C) AlexNet.

The training and testing of these architectures phases were carried out using different batch sizes and learning rates to identify which of the proposed architectures offered the best classification results (hyper-parameter search). Figure 8 shows the accuracy of each of the proposed architectures for a batch size of 20 and a learning rate of 0.001. As demonstrated, the lowest performance resulted when using the LeNet-5 architecture (Fig. 8A). On the contrary, the best performance indicators could be noted using the VGG and the AlexNet architectures (Figs. 8B and 8C). Nevertheless, as illustrated in Fig. 9, the VGG16 architecture reveals premature overfitting. Overfitting can be observed in the VGG16 architecture almost from the beginning of the training, around epoch 20 (Fig. 9B). In the case of the AlexNet architecture, overfitting started around epoch 40 (Fig. 9C). These findings suggest that, under these parameters, AlexNet has a better capacity of generalization than its competitors.

**Figure 10:**
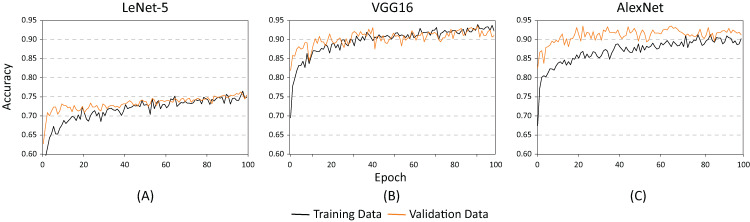
Accuracy results for batch size = 100 and learning rate = 0.001 for (A) LeNet-5, (B) VGG16, and (C) AlexNet.

**Figure 11:**
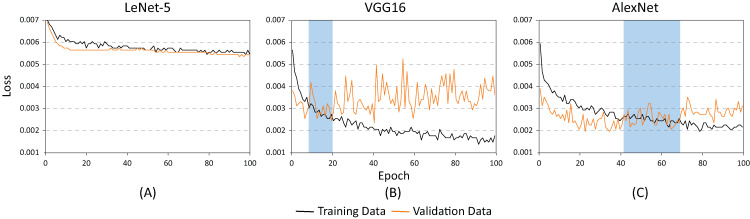
Loss results for batch size = 100 and learning rate = 0.001 for (A) LeNet-5, (B) VGG16, and (C) AlexNet.

Figures 10 and 11 show the results obtained when adjusting the batch size from 20 to 100. As illustrated in Fig. 10, the behavior of the models is similar to the results presented in Fig. 8. However, one main difference can be observed in Fig. 10. In the case of VGG16 (Fig. 10B), the increase in batch size had a negative impact because the overfitting problem occurred practically at the beginning of the training process. In the case of the AlexNet architecture (Fig. 10C), the impact is considered positive since it allows the overfitting to occur later. This has a effect on the model’s capability of generalization. When comparing Figs. 8 and 10, it is clearly proven that AlexNet gained 20 additional training epochs. Therefore, it is possible to conclude that the increase in batch size enhanced AlexNet’s generalization ability by preventing overfitting.

Table 2 shows the basic statistical results obtained during the training process of the three models. As demonstrated, the architectures with the highest accuracy rates are the AlexNet and the VGG16. However, as illustrated in Figs. 9 and 10, the VGG16 is more likely to present overfitting.

**Table 2:** Accuracy rate.

Model	Max(%)	Min(%)	Mean(%)
AlexNet	93.54	82.50	91.09
VGG16	93.54	81.88	90.37
LeNet-5	76.67	63.13	73.64

After analyzing the behavior of the different neural networks studied so far, and modifying some of their hyper-parameters, findings from this study indicate that the AlexNet architecture demonstrated the best performance. In consequence, AlexNet exhibited the best capacity for generalization while maintaining the lowest overfitting. As shown in Fig. 10, the AlexNet neural network does not begin to present overfitting behaviors until the 40’s epoch. With all this information, we proceeded to retrain the AlexNet neural network, using the following parameters: learning speed of 0.001, batch size of 100, and 40 training periods.

**Figure 12:**
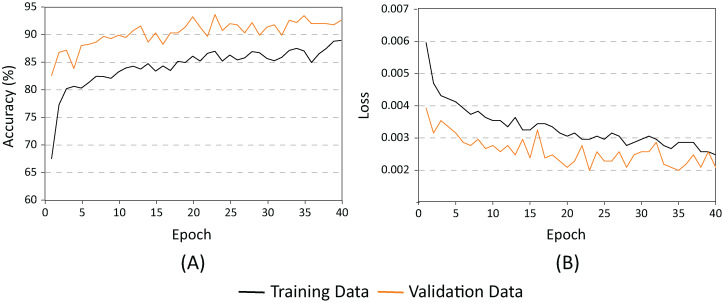
AlexNet for batch = 100 and learning rate = 0.001. (A) Accuracy and (B) Loss.

Figure 12 shows AlexNets’ evolution during the training period. As demonstrated, there is no indication of overfitting. Evidently, the capacity of generalization of the final model is superior to those shown previously. The accuracy of the model (Fig. 12A), using the evaluation dataset consistently improved the results with the training dataset. It is also clear that because the AlexNet loss (Fig. 12B) for the evaluation dataset remained below the test dataset, the accuracy achieved by the network during training is of 94%.

**Figure 13:**
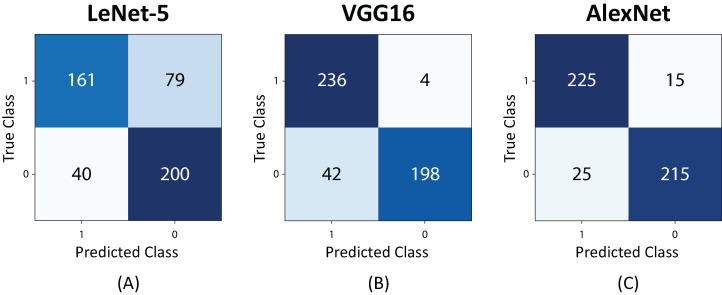
Confusion Matrix for 480 test images. (A) LeNet-5, (B) VGG16, and (C) AlexNet.

Figure 13 shows the confusion matrices for the studied architectures. The highest number of false positives and false negatives were obtained by the LeNet-5 neural network. In the case of the VGG16, a bias was observed when the network confused images with and without *Sargassum* in a high proportion. The AlexNet presented a less biased and more balanced behavior, resulting in a neural network with a higher capacity of generalization.

Finally, Table 3 demonstrates the precision, recall, F1-score, and support results for the AlexNet. It is clearly evidenced that the evaluation dataset is balanced. The results show a maximum recall of 94% and an f1 score of 92%. This is considered a significant result considering that the AlexNet neural network training was performed with a relatively small set of balanced images.

**Table 3:** AlexNet classification results.

	Precision (%)	Recall (%)	F1-score (%)	Support
*Sargassum* absence	90	94	92	240 images
*Sargassum* presence	93	90	91	240 images
Accuracy			92	480 images

In the case of the *Sargassum* monitoring along the beaches, it is important that service providers, tourists, visitors, and authorities in charge of beach sanitation know where it accumulates for decision-making processes. There is evidence in social networks (https://www.viajefest.com/sargazo-en-quintana-roo/), of some citizen initiatives proposing maps that indicate the presence of *Sargassum* along the coast. However, because these maps are hand-designed, they present some disadvantages. Some of these include a quick expiration and the lack of measures to verify the accuracy or reliability of the sources used for map generation. Therefore, a next step followed was to classify the geotagged images from the Collective View platform to automatically generate maps showing the presence or absence of *Sargassum* along the beaches. As far as we know, the present study proposes the first automatic system to generate a *Sargassum* presence or absence map that helps society manage high concentrations of *Sargassum* along the beaches.

**Figure 14:**
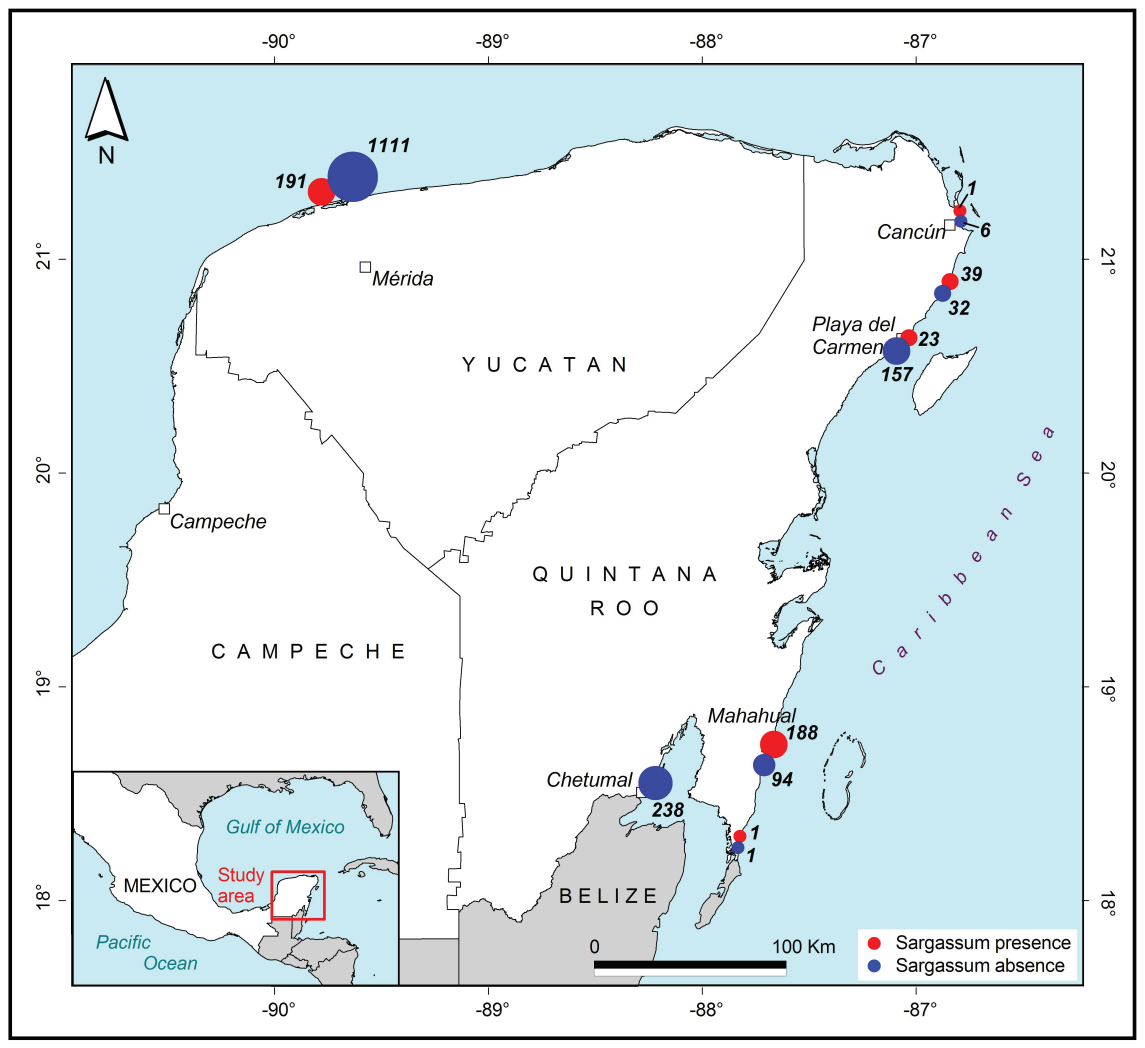
*Sargassum* distribution map. *Sargassum* distribution map built with the geotagged photographs previously classified, collected through crowdsourcing.

**Figure 15:**
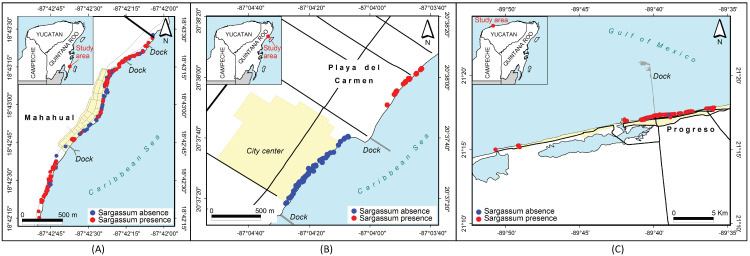
*Sargassum* distribution map along the beaches of three cities. *Sargassum* distribution map along the beaches of three cities, built with the geotagged photographs previously classified, collected through crowdsourcing. (A) Mahahual, Q. Roo, (B) Playa delCarmen, Q. Roo and (C) Puerto Progreso, Yucatan.

The first approximations of maps that have been generated from previously classified images are shown in Figs. 14 and 15. Photographs classified with and without the presence of *Sargassum*, and grouped by region and cities, along the coast in the Yucatan Peninsula are shown in Fig. 14. The images were taken between August 15 and December 18, 2019. The red dots represent the presence of *Sargassum* in the images, and the blue dots represent the absence of *Sargassum*.

Figure 15 allows for a closer examination of the beaches in three cities in the region, namely Mahahual (Fig. 15A), Playa del Carmen (Fig. 15B), and Puerto Progreso (Fig. 15C). In this case, the position of each point represents the place where the photograph was taken. Again, the red dots represent the presence of *Sargassum* in the images, and the blue dots represent the absence of *Sargassum*. The photographs in the maps in Fig. 15 were taken on different days. The analysis of each situation required adding information such as the time and date the photo was taken, if the area was periodically cleaned, among other factors. As an example, in Mahahual, most of the photographs were taken between September 14 and 15. This is a vacation weekend at the end of the *Sargassum* arrival season, and as demonstrated, the *Sargassum* density is highest in the north and south of the hotels and restaurants area, which suggests that service providers cleaned their beaches to improve their service to tourists. In the case of Playa del Carmen, the photographs in front of the City Center were taken between November 28 and 29, when the arrival season for *Sargassum* had already ended. The photographs northeast of the City Center were taken between August 21 and 22, during the arrival season. Finally, in the case of Puerto Progreso, the map shows points that correspond to photographs taken between August 15 and December 18, 2019. Thus, to see the detail by month, week, or day, additional maps are required to the study date period. The economic activities are more diverse in Puerto Progreso than in Mahahual or Playa del Carmen, so the presence of *Sargassum* in Progreso is not as critical. Therefore, the beaches are not cleaned as frequently as in the other sites.

The maps demonstrating the presence or absence of *Sargassum* are a first approximation of what is possible to achieve with data obtained from crowdsourcing. The information exhibited in Figs. 14 and 15 reflect this initial approximation. It is known that *Sargassum* monitoring is dynamic in time, and that these maps represent the distribution of *Sargassum* between August 15 and December 18 in 2019. Therefore, further research should explore generating automatized maps by uploading daily pictures to the platform. However, achieving this goal requires a constant flow of information. Another area for further research could be to develop methods to assess the coverage of the *Sargassum* within the images, and label them based on the amount of *Sargassum* in the area (e.g., excessive, a lot, or little). As a validation of the methodology, we consider that it has been a success; however, we also know that the basis of this proposal relies on constant citizen participation.

## Conclusions

*Sargassum* monitoring through traditional satellite remote sensing is not always effective due to various factors. Some limitations that traditional methods cannot account for include: environmental conditions in the region (e.g., high humidity and the presence of clouds); the conditions of the coastline which have been mentioned above; as well as the spatial and temporal limitations of the satellite platforms. Therefore, we consider this study proposes a significant and viable addition to satellite monitoring.

Supported by a crowdsourcing application called Collective View, which was developed by the authors and with the contribution of members of society, this study offers the largest set of geotagged images in the world for the northern region in the Yucatan Peninsula and the Mexican Caribbean. At the time of this study, there were 4035 photographs in the platform. Additionally, using augmented data and knowledge transfer, the AlexNet neural network was trained and reached a maximum recall of 94% and an f1 score of 92%. This can be considered a good result considering that the training was performed with a relatively small set of balanced images.

Through the use of an automatic classification of geotagged images, the present study proposes an automatic system to generate *Sargassum* presence and/or absence maps that support society to manage high concentrations of *Sargassum* along the beaches in the Yucatan Peninsula. To the best of our knowledge, this is the first proposal of its kind for this region. For the continuous updating of the maps, it is necessary to capture daily images from different zones and classify them automatically. In this way, it will be possible to take advantage of this technology for the benefit of service providers, authorities involved in the sanitation of beaches, as well as tourists, and visitors. The constant participation of society, as a whole, is required for the full use of current technologies.

Regarding *Sargassum* observation, important efforts have been implemented to monitor pelagic *Sargassum* and other macroalgal abundance and distribution. However, these efforts use a wide variety of methods that are often not comparable. We believe that it is necessary to build a common platform to facilitate communication and collaboration regarding this issue. In this sense, combining crowdsourcing, current communication technologies, and Artificial Intelligence techniques like neural networks and computer vision, are a viable option to build maps on a global and regional scale in near real-time. Generating maps automatically requires an instant classification of the collected information. Thus, automatic classification becomes relevant.

The operation of the crowdsourcing platform remains a technological challenge. Nevertheless, in our experience, the main challenge of crowdsourcing was to maintain the permanent involvement of society members throughout the study. A more difficult challenge posed is for society to adopt crowdsourcing as a useful and routine habit in this constantly changing digital civilization.

One of the goals of our future work involves completing and exploiting the *Sargassum* dataset. Although we achieved our initial goal of creating the largest dataset in the world of geotagged images for the coasts of Quintana Roo, crowdsourcing also allows for images from different countries around the Caribbean Sea to be entered. In fact, the dataset currently has some images of Guadeloupe and Florida. Thus, an ongoing challenge is that the geotagged dataset continues to grow with images from different places and dates.

A second goal of our future work is related to the coverage of *Sargassum* within images. As shown throughout this work, images were classified in two categories: images with and without *Sargassum*. This is an arduous process that requires the use of various techniques to be performed successfully. As part of our future work, we will try to determine the coverage of the *Sargassum* within the images, developing a method that allows an effective semantic segmentation of the images to measure the amount of *Sargassum* present on the beaches. This will be supported by machine learning techniques to create models that allow us to accurately estimate the coverage of this macro-algae along the beaches in Quintana Roo.

## Supplemental Information

10.7717/peerj-cs.528/supp-1 Supplemental Information 1 Source code for Jupyter Notebook.

## Additional Information and Declarations

### Data Availability

The following information was supplied regarding data availability: Data for the Neural Networks training is available at figshare: Arellano-Verdejo, Javier; Lazcano-Hernandez, Hugo E. (2020): Sargassum Dataset. figshare. Dataset. DOI 10.6084/m9.figshare.13256174.v5.
